# High-level expression, purification, and enzymatic characterization of truncated human plasminogen (Lys531-Asn791) in the methylotrophic yeast *Pichia pastoris*

**DOI:** 10.1186/s12896-015-0179-z

**Published:** 2015-06-09

**Authors:** Rongzeng Liu, Bing Zhao, Yanling Zhang, Junxiang Gu, Mingrong Yu, Houyan Song, Min Yu, Wei Mo

**Affiliations:** Key Laboratory of Metabolism and Molecular Medicine, Ministry of Education, Fudan University, 138 Yixueyan Rd, Shanghai, 200032 China; Department of Biochemistry and Molecular Biology, School of Basic Medical Sciences, Fudan University, 138 Yixueyan Rd, Shanghai, 200032 China; Collaborative Innovation Center for Biotherapy, Sichuan University, Huaxi Campus: No.17 People’s South Road, Chengdu, 610041 China

**Keywords:** Truncated plasminogen, Plasmin, *Pichia pastoris*, Purification, rhμPlg

## Abstract

**Background:**

Plasmin is a serine protease that plays a critical role in fibrinolysis, which is a process that prevents blood clots from growing and becoming problematic. Recombinant human microplasminogen (rhμPlg) is a derivative of plasmin that solely consists of the catalytic domain of human plasmin and lacks the five kringle domains found in the native protein. Developing an industrial production method that provides high yields of this protein with high purity, quality, and potency is critical for preclinical research.

**Results:**

The human microplasminogen gene was cloned into the pPIC9K vector, and the recombinant plasmid was transformed into *Pichia pastoris* strain GS115. The concentration of plasmin reached 510.1 mg/L of culture medium. Under fermentation conditions, the yield of rhμPlg was 1.0 g/L. We purified rhμPlg to 96 % purity by gel-filtration and cation-exchange chromatography. The specific activity of rhμPlg reached 23.6 U/mg. The *K*_*m*_ of substrate hydrolysis by recombinant human microplasmin was comparable to that of human plasmin, while rhμPlm had higher *k*_*cat*_/*Km* values than plasmin. The high purity and activity of the rhμPlg obtained here will likely prove to be a valuable tool for studies of its application in thrombotic diseases and vitreoretinopathies.

**Conclusions:**

Reliable rhμPlg production (for use in therapeutic applications) is feasible using genetically modified *P. pastoris* as a host strain. The successful expression of rhμPlg in *P. pastoris* lays a solid foundation for its downstream application.

**Electronic supplementary material:**

The online version of this article (doi:10.1186/s12896-015-0179-z) contains supplementary material, which is available to authorized users.

## Background

Plasminogen, the proenzyme precursor of the primary fibrinolytic protease, plasmin, is a single-chain protein containing of 791 amino acid residues [1]. While plasminogen is expressed in all major organs and tissues, it is synthesized primarily in the liver [2]. Plasminogen is found in significant quantities in extravascular fluids [3]. Circulating plasminogen comprises a Pan-apple (PAp) domain, five kringle domains (KR1–5), and a serine protease (SP) domain [4]. Under physiological conditions, plasminogen is converted to plasmin by a cleavage event that occurs in its activation loop (between Arg561 and Val562), which is facilitated by tissue plasminogen activator (tPA) or urokinase plasminogen activator (uPA). The plasmin molecule is a two-chain, disulfide-linked SP with trypsin-like specificity. The C-terminal light chain of plasmin (residues 562–791, molecular weight (MW) 25 kDa) contains a typical SP that is homologous to trypsin. The C-terminal light chain also contains the classic catalytic triad, which is composed of His603, Asp646, and Ser741.

Plasmin can undergo an autolytic process, and its cleavage site specificity can change with pH. The specific autolytic cleavage of the plasmin molecule in alkaline solutions leads to the formation of a low-molecular-weight form of plasmin, termed microplasmin [5]. Human microplasmin (Lys531–Asn791), which was first prepared from plasmin in an alkaline solution, consists of two polypeptide chains that are connected by disulfide bonds. One polypeptide is the B chain of plasmin that consists of 230 amino acids, while the other peptide is the C-terminal portion of the A chain of plasmin, which consists of 31 amino acid residues [6]. Microplasmin has a molecular weight of 28 kDa, as calculated from its primary sequence. It is slightly more positively charged than plasminogen, and it is generally a more hydrophobic molecule [6].

The dissolution of blood clots, whether accomplished physiologically or pharmacologically, is facilitated by plasmin [7]. Plasmin was first studied more than 50 years ago, and it was found to be either ineffective or inferior to plasminogen activators in treating thrombotic diseases [8]. With the current widespread use of catheters to deliver thrombolytic therapy, plasmin has been reevaluated as a potentially valuable thrombolytic agent; pharmacological studies have demonstrated plasmin is an effective and hemostatically-safe application for the treatment of thrombotic diseases [9]. In addition to fibrin, plasmin has been shown to directly degrade other extracellular matrix (ECM) components, including laminin and fibronectin, which have a postulated role in vitreoretinal adhesion [10, 11]. In both enucleated pig and human eyes, the addition of 1 U of plasmin eliminates the cortical vitreous remnants previously detected on the inner limiting membrane, all without the use of adjunctive techniques [12, 13].

However, the isolation of autologous plasmin is a costly and time-consuming process. Furthermore, it is difficult to produce plasmin via recombinant gene technology because of its high molecular weight and tendency to degrade [14]. Therefore, the availability of lower molecular weight and more stable microplasminogen (μPlg) represents a valuable tool that might be applied to the treatment of thrombotic diseases and vitreoretinopathies (pending preclinical and clinical evaluation). In response to this need, we developed a protocol to produce abundant quantities of highly active and high-purity rhμPlg (Lys531–Asn791) from *Pichia pastoris*.

## Results

### Construction of plasmid pPIC9K-*hμPlg*

A full-length DNA sequence of the synthetic *hμPlg* gene was inserted into the open reading frame of the α-factor signal sequence of the *P. pastoris* expression vector pPIC9K, which is under the regulation of the *AOX1* promoter (Additional file 1). This plasmid was transformed into *Escherichia coli* DH5α to express hμPlg. Enzyme digestion demonstrated that the *hμPlg* gene was correctly oriented in the pPIC9K vector (Additional file 1). DNA sequencing of the recombinant vector confirmed the accuracy of the reading frame of *hμPlg*, and *P. pastoris* GS115 positive transformants were successfully identified. The plasmid pPIC9K-*hμPlg* contained a 783-bp inserted target fragment. The deduced mature cDNA sequence of *rhμPlg* encodes a 261-amion acid peptide with a theoretical MW of 28.63 kDa and a p*I* of 7.95.

### Transformation and screening of transformants

After linearization with *Sal*I, pPIC9K-*hμPlg* was transformed into *P. pastoris* GS115 competent cells by electroporation. After an initial selection of His + transformants, clones were grown in microtiter plates until they reached the same optical density. The cultures were then spotted onto yeast extract-peptone-dextrose (YPD)-Geneticin plates (containing 2.0 mg/mL of Geneticin) and scored for Geneticin resistance. Five transformants, B2, B6, C3, F2, and F6, were obtained on the YPD-Geneticin plates. After genomic DNA was isolated from *P. pastoris* recombinants and controls, polymerase chain reaction (PCR) verification of the positive recombinants was performed using 5′ and 3′ *AOX1* sequencing primers. Two bands were obtained for pPIC9K-*hμPlg*: one corresponding to the size of the *hμPlg* gene plus the inserted *AOX1* gene in pPIC9K (783 + 492 bp), and the other corresponding to the *AOX1* gene in the chromosome of *P. pastoris*.

### Expression of rhμPlg in a shaker flask and a 5-L bioreactor

**Fig. 1:**
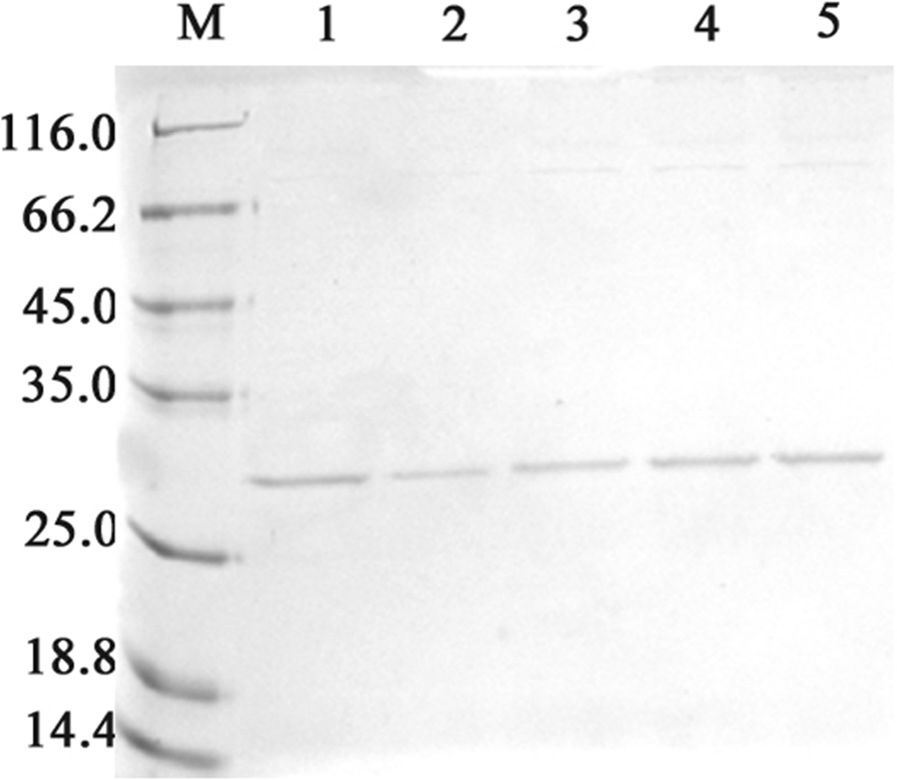
Production of rhμPlg in *P. pastoris* GS115 using a shaker flask. This figures shows the SDS-PAGE analysis of rhμPlg in fermentation supernatants from different transformants induced for 96 h in shaker flask cultures. Lanes 1–5A are loaded with 20 μl of fermentation supernatants from different transformants; lane M contains 10 μl of molecular mass standards

The selected transformants (B2, B6, C3, F2, and F6) were cultured in a shaker flask to measure rhμPlg expression. Maximum expression was observed after 4 days of methanol induction at 30 °C. Supernatant proteins were subjected to sodium dodecyl sulfate–polyacrylamide gel electrophoresis (SDS-PAGE) analysis (Fig. 1, lanes 1–5). As shown in Fig. 1, a predominant band at approximately 35 kDa was observed in lanes 1–5. No target band was detected in the cultures of an induced transformant harboring an empty pPIC9K plasmid. GS115 F2 showed a distinctive band (lane 5), with a yield that reached 510.1 mg/L after 96 h of induction. Therefore, GS115 F2 was used for the subsequent expression of rhμPlg.

**Fig. 2:**
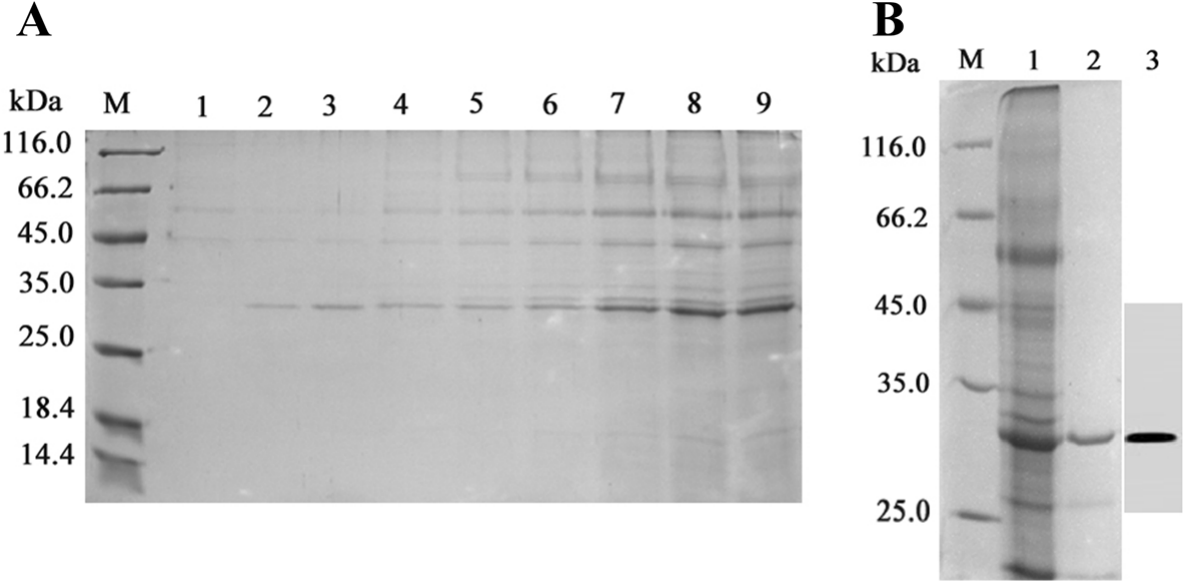
Expression and purification of rhμPlg. **a**, SDS-PAGE analysis of rhμPlg expressed by fed-batch fermentation. Lane 1, standard protein marker (14.4–116.0 kDa); Lane 1, uninduced *P. pastoris*; Lanes 2–9, *P. pastoris* induced with methanol for 5, 10, 15, 20, 25, 30, 35, and 40 h. **b**, M, standard protein marker. Lane 1, gel-filtration chromatography wash fraction; Lane 2, cation-exchange chromatography wash fraction 3; Lane 3, western blot of rhμPlg

As shown in Fig. 2a, fermentation was conducted in a 5-L bioreactor. The culture medium was collected and centrifuged every 5 h to obtain supernatants. Recombinant protein expression started 5 h after methanol induction and reached a peak at 40 h (Fig. 2a). At the end of the glycerol fed-batch phase, the biomass concentration reached 90 g/L. The methanol fed-batch phase of rhμPlg production lasted for 40 h, and the final biomass concentration reached 190 g/L, while the volume of the supernatant increased to 3.4 L. After 40 h of induction, the total secreted protein concentration was 1.88 g/L, and the rhμPlg activity in the supernatant reached 7.9 U/mL, which was 15 times that of the shaker flask fermentation.

### Purification of rhμPlg

The concentration of rhμPlg in the bioreactor culture supernatant was estimated to be 1.0 g/L based on SDS-PAGE. The rhμPlg expressed by *P. pastoris* was purified to homogeneity by ultrafiltration, followed by cation-exchange and Superdex 75 gel-filtration chromatography. The culture supernatant was collected and concentrated using membrane ultrafiltration. Subsequently, concentrated supernatant was loaded onto an SP Sepharose Fast Flow column, which bound most of the rhμPlg (Fig. 2b). rhμPlg was eluted at 0.35 mol/L in NaCl-acetate buffer. rhμPlg was further purified via high-resolution gel-filtration chromatography to remove trace impurities. Finally, we obtained 167 mg of purified rhμPlg per liter of culture supernatant (Additional file 2). The specific activity of purified rhμPlg reached 23.6 U/mg protein (Additional file 2). The eluates containing rhμPlg were analyzed using SDS-PAGE, followed by western blotting using a polyclonal antibody. The purity of rhμPlg reached 96 %, as determined by SDS-PAGE (Fig. 2b, lane 2).

### Characterization of rhμPlg

N-terminal sequencing indicated that the 10 amino acid residues at the N-terminus of rhμPlg were KLYDYCDVPQ, which matched the deduced amino acid sequence of mature hμPlg. We determined that the secreted active protein contained 261 amino acids, which is consistent with the size of full-length hμPlg.

### Mass spectrometry analysis

**Fig. 3:**
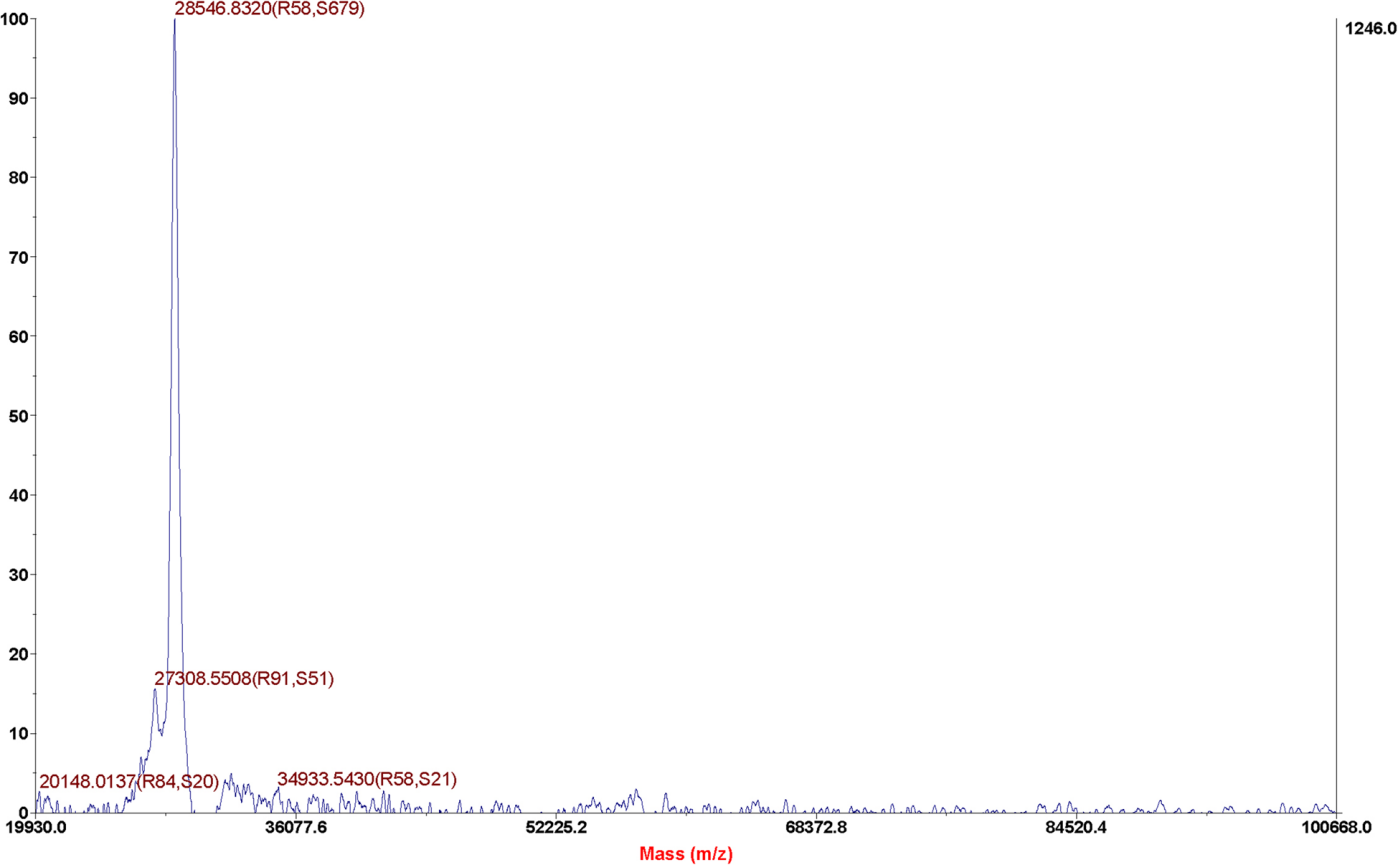
Molecular mass determination of purified rhμPlg by LC-MS. Peaks corresponding to various protonated rhμPlg species are marked. The mass analysis determined that rhμPlg has a molecular weight of 28.55 kDa

An accurate mass can be detected by intact mass analysis using liquid chromatography-mass spectrometry (LC-MS). Peaks corresponding to various protonated rhμPlg species were determined. The mass analysis determined that rhμPlg has a molecular weight of 28.55 kDa (Fig. 3). This is identical to that identified by SDS-PAGE.

### Amidolytic assays and kinetic analysis

**Fig. 4:**
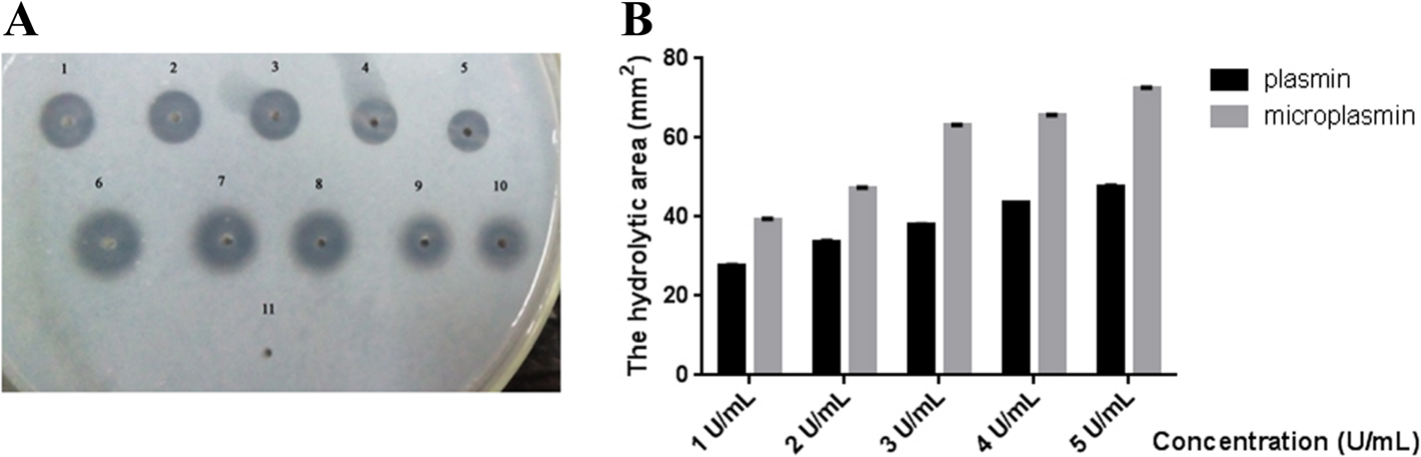
Fibrinolytic activity of rhμPlg and plasminogen in a plasminogen-free fibrin agar plate. **a**, 1**–**5, plasmin with a concentration of 5, 4, 3, 2, and 1 U/mL, respectively; 6–10, rhμPlg with a concentration of 5, 4, 3, 2 and 1 U/mL, respectively; 11, PBS as negative control. **b**, Comparison of the fibrinolytic activity of plasmin and rhμPlg as calculated from the area of the lysis zone

Both plasmin and rhμPlg possess amidolytic activity against chromogenic substrates. The kinetic parameters *K*_*m*_, *k*_*cat*_, and *k*_*cat*_/*K*_*m*_ for such substrates are summarized in Table 1. The *K*_*m*_ of substrate hydrolysis by rhμPlg was comparable to that by human plasmin (Table 1), suggesting that kringle domains 1–5 had no effect on the catalytic activity of plasmin. We also found that rhμPlg had higher *k*_*cat*_/*K*_*m*_ values than plasmin. The difference in the *k*_*cat*_/*K*_*m*_ values of the two proteinases was caused mainly by a variance in *k*_*cat*_ (Table 1), which implied that there was a structural difference between the two proteins, probably owing to the closed conformation of plasmin. In addition, both serine proteases hydrolyzed a fibrin plate in a concentration-dependent manner, with slightly higher area values for rhμPlg (Fig. 4).

**Table 1:** Kinetic parameters: comparison of plasminogen and rhμPlg

Enzyme species	Amidase parameters		
	*K*_*m*_ (mM)	*k*_*cat*_ (S^−1^)*	*k*_*cat*_/*K*_*m*_ (mM^−1^ · S^−1^)*
Plasminogen	0.325 ± 0.016	30.6 ± 2.5	94.15 ± 0.03
rhμPlg	0.348 ± 0.021	44.5 ± 3.6	127.87 ± 0.01

Values given are means ± SEM (*n* = 3); *P values < 0.05

## Discussion

Plasminogen and its active form (plasmin) play important physiological and pathological roles in fibrinolysis and hemostasis, the degradation of the ECM, cell migration, tissue remodeling, wound healing, angiogenesis, inflammation, and tumor cell migration [15]. Consequently, patients with severe plasminogen deficiency typically suffer from a range of difficult to treat inflammatory conditions, including ligneous conjunctivitis, gingivitis, and neural disorders [16, 17]. Activation of the plasminogen system is tightly regulated, which is necessary to prevent the development of a systemic fibrinolytic state or tumor metastasis. The availability of rhμPlg with full enzymatic activity could be extremely valuable for structure-function studies of plasminogen and plasmin. In addition, its truncated derivative is also needed for various important therapeutic applications, including catheter-directed thrombolysis and treatment of acute ischemic stroke [18], acute lower extremity arterial or bypass graft occlusion [19], acute iliofemoral deep venous thrombosis [20] enzyme-induced vitreolysis, alleviating the progression of diabetic retinopathy [21], and treatment of diabetic retinopathy in young adults [22].

rhμPlg has been expressed at high levels as inclusion bodies in *E. coli* [23, 24]. Recently, the methylotrophic yeast *P. pastoris* has been considered to be an excellent host for producing proteins obtained from different sources [25]. As a eukaryote, *P. pastoris* has many advantages. Proteins produced by *P. pastoris* are more likely to be processed, properly folded, and chemically modified. High expression levels, high cell densities in culture, easier upscaling, and strong and tightly regulated promoters have all been identified as advantages of using *P. pastoris* as a host [26]. In this study, rhμPlg was expressed in *P. pastoris*, which was achieved by expressing rhμPlg (comprising amino acids Lys531–Asn791), which lacks the five kringle domains. This method produced high yields (1.88 g/L of fermentation broth) of protein, which could be purified to homogeneity. The MW of rhμPlg was different from that of the microplasmins produced by Nagai et al. and Joshi et al. [27, 28], both of which lacked 12 N-terminal amino acids.

In this study, we cloned the cDNA gene of *hμPlg* into a pPIC9K vector that contained an α-factor secretion signal and allowed for the expression of hμPlg in *P. pastoris* GS115. Transformants were selected based on their ability to grow on a medium lacking histidine, as well as on their Geneticin resistance. Enzymatically active rhμPlg, which had a MW of 28.55 kDa, was expressed. Then, we developed a preparation of rhμPlg that was suitable for pharmaceutical use. The final, optimized process consists of a sequence of three chromatography steps. The first step (gel filtration) desalts and exchanges the buffer to acetate buffer, while the second step (cation exchange) captures rhμPlg and allows contaminants to flow through. A gel filtration/diafiltration step then serves to transfer the recombinant protein into its formulation buffer (20.0 mM phosphate buffer, pH 7.0). The final concentration was 1 mg/ml in phosphate-buffered saline (pH 7.0). A preliminary characterization of rhμPlg was performed using western blotting, high-performance liquid chromatography, mass spectrometry, and N-terminal sequencing to verify the identity of the protein. We also completed a partial characterization of the functional properties of rhμPlg by comparing its serine protease activity to that of commercially available plasmin. Our assays showed that when using an amidolytic chromophogenic substrate, the *K*_*m*_ value of rhμPlg was similar to that of plasmin (Fig. 4). The catalytic efficiency, *k*_*cat*_/*K*_*m*_, was higher for rhμPlg than plasmin. rhμPlg, at a concentration similar to that of plasmin, exhibited a higher area value in the fibrinolysis plate, suggesting that kringle domains 1–5 have no effect on the catalytic activity of plasmin.

The development of rhμPlg may solve most of the problems associated with the treatment of vitreoretinopathies with autologous plasmin. rhμPlg contains only the enzymatic portion of the plasmin molecule, while the other domains are omitted. This makes rhμPlg much smaller than the original molecule (28 vs. 80 kDa, respectively) while retaining the same enzymatic activity, which theoretically would allow for greater penetration into epiretinal tissues. rhμPlg has other advantages over plasmin. First, it is commercially available, allowing investigators to avoid the time-consuming and expensive steps necessary for the production of autologous plasmin enzyme. Second, it is generated by recombinant techniques, ensuring its sterility while avoiding the risk of microbial contamination. Last, it is inherently more stable than plasmin and can be stored in citrate buffer prior to use [29].

## Conclusions

Our results demonstrate that high-purity rhμPlg (Lys531–Asn791) can be abundantly produced by *P. pastoris*. After purification and identification of rhμPlg, we observed fibrinolytic activity in vitro, which suggested that rhμPlg might play an active and beneficial role in treating thrombotic diseases and controlling the progression of vitreoretinopathies. Therefore, the possibility of producing rhμPlg for use in therapeutic applications could be fulfilled by *P. pastoris*. The successful expression of rhμPlg in *P. pastoris* lays a solid foundation for its possible future applications.

## Methods

### Strains, vectors, reagents, and enzymes

The multi-copy *Pichia* expression kit containing *P. pastoris* strain GS115 and the pPIC9K vector were purchased from Invitrogen Corp. (Carlsbad, CA, USA). The *E. coli* strain DH5α was purchased from Novagen (EMD Millipore, Billerica, MA, USA). *E. coli* cells containing plasmids were cultured aerobically at 37 °C in Luria–Bertani medium (5 g/L yeast extract, 10 g/L tryptone, 10 g/L NaCl, and 15 g/L agar) containing 100 μg/ml ampicillin to maintain the plasmids. Minimal dextrose (MD) medium, buffered complex glycerol medium (BMGY), YPD medium, buffered complex methanol medium (BMMY), and fermentation basal salts medium (BSM) supplemented with PTM_1_ solution (0.2 g/L H_3_BO_3_, 6.0 g/L CuSO_4_^.^5H_2_O, 0.8 g/L KI, 3.0 g/L MnSO_4_^.^2H_2_O, 65 g/L FeSO_4_^.^7H_2_O, 0.2 g/L Na_2_MoO_4_^.^2H_2_O, 0.5 g/L CoCl_2_, 20.0 g/L ZnCl_2_, 5 mL/L H_2_SO_4_, 0.5 g/L CaSO_4_^.^H_2_O, and 0.2 g/L biotin) were prepared according to Invitrogen’s instructions for *P. pastoris* fermentation. Restriction enzymes and kits for cloning were obtained from New England Biolabs (Ipswich, MA, USA). The gene of interest, obtained from NCBI (GenBank: M74220.1, human plasminogen mRNA, complete coding sequence, nucleotides 1648–2430) was synthesized and cloned into plasmid pUC57 by Sangon Biological Co., Ltd. (Shanghai, China) (Additional file 3). The chromogenic substrate H-D-Val-Phe-Lys-p-NA · 2HBr (S2390) was synthesized by the School of Pharmacy, Fudan University [30].

### Synthesis of the human μPlg gene and construction of the expression vector

A DNA fragment was amplified by PCR using the synthetic gene as a template, which yielded an 814-bp product. The forward primer was 5′-CCG*CTCGAG*AAAAGAAAACTTTACGACTACTGTG (the underlined sequence is an *Xho*I site), and the reverse primer was 5′-ATAAGAAT*GCGGCCGC*TTAATTATTTCTCATCACTCC (the underlined sequence is a *Not*I site). The total reaction volume was 20 μL, including 4 μL of 5× Phusion HF DNA polymerase, 0.4 μL of 10 mM dNTPs, 1.0 μL of the forward primer (10 mM), 1.0 μL of the reverse primer (10 mM), 0.25 μL of Phusion DNA polymerase, 12.35 μL of sterile water, and 1 μL of pUC57-μPlg (10 ng/μL). Amplification conditions were 30 s at 98 °C, 30 cycles of 10 s at 98 °C, 20 s at 49 °C, and 30 s at 72 °C, followed by a final extension of 5 min at 72 °C. The PCR product was ligated into the pPIC9K vector between the *Xho*I and *Not*I sites. The recombinant plasmid was transformed into *E. coli* JM109, and transformants were identified by restriction digest analysis and sequencing. The theoretical MW and p*I* were calculated using the website http://web.expasy.org/compute_pi/.

### Transformation and screening of multi-copy transformants

*Sal*I was used to linearize 10 μg of the pPIC9K-*hμPlg* plasmid, and the linear DNA was then transformed into *P. pastoris* GS115 cells using a GenePulser system (Bio-Rad, Carlsbad, CA, USA) under the following conditions: 1.5 kV, 200 Ω, and 25 μF). His + transformants were selected on YPD plates containing Geneticin 418 (2–6 mg/ml), and then lysed by a combined enzyme, freezing, and heating treatment according to a simple protocol reported in the literature [31]. The cell lysate containing the genomic DNA was analyzed by PCR using the 5′ *AOX1* primer 5′-GACTGGTTCCAATTGACAAGC-3′ and the 3′ *AOX1* primer 5′-GCAAATGGCATTCTGACATCC-3′. The total reaction volume was 50 μL, including 5 μL of 10× reaction buffer, 5 μL of 25 mM MgCl_2_, 1 μL of 25 mM dNTPs, 1 μL of the 5′ *AOX1* primer (10 pmol/μL), 1 μL of the 3′ *AOX1* primer (10 pmol/μL), 27 μL of sterile water, and 5 μL of cell lysate. The reaction was incubated in a thermocycler at 95 °C for 5 min, and 5 μl of a 0.16 U/μl solution of *Taq* polymerase (0.8 units) was added. The amplification conditions were 30 cycles of 1 min at 95 °C, 1 min at 54 °C and 1 min at 72 °C, followed by a final extension of 7 min at 72 °C.

### Production of rhμPlg in a shaking flask and a 5-L bioreactor

The colonies of His + transformants exhibiting resistance to 2.0 mg/ml Geneticin were inoculated in 10 ml of BMGY (2 % peptone, 1 % yeast extract, 100 mM potassium phosphate, pH 6.0, 1.34 % yeast nitrogen base (without amino acids), 0.4 μg/ml biotin, and 1 % glycerol) at 30 °C with constant shaking at 250 rpm until the OD_600_ was 4. To accurately measure OD_600_ > 1.0, a sample of the culture was diluted 10-fold before reading. The cells were then resuspended in 10 ml of BMMY (2 % peptone, 1 % yeast extract, 100 mM potassium phosphate, pH 6.0, 1.34 % yeast nitrogen base (without amino acids), 0.4 μg/ml biotin, and 0.5 % methanol), and expression was induced at 30 °C with constant shaking at 250 rpm for 96 h. Methanol was added at a concentration of 1 % (v/v) every 24 h. The supernatant was collected for activity assays and SDS-PAGE analysis.

The inoculum seeds of *P. pastoris* were cultured at 30 °C on a shaker at 250 rpm for 24 h in 200 ml of YPD medium. A total of 200 ml of the seed broth was inoculated into a 5-L fermenter (Bioflo 3000 fermenter, New Brunswick Scientific, New Brunswick, NJ, USA) containing 3 L of BSM plus 2 ml/L of PTM_1_ solution. The pH of the medium was adjusted to and maintained at 5.0 via the addition of ammonium hydroxide. The temperature was maintained at 30 °C, and the dissolved oxygen level was maintained at over 35 % of air saturation by a cascaded control of agitation speed and aeration rate. After glycerol exhaustion, 200 ml of feeding medium containing 50 % (w/v) glycerol and 2 ml/L of PTM_1_ solution were pumped into the bioreactor according to Invitrogen’s instructions for *P. pastoris* fermentation. After the depletion of glycerol, pure methanol containing 2 ml/L of PTM_1_ was added to the bioreactor. The production phase lasted 40 h at 30 °C, with a gradual increase in the methanol feeding rate from 0.8 to 4 ml/L.h, which allowed the culture to adapt to the methanol. After 6 h, the methanol feed rate was maintained at 4 ml/L/h for an additional 34 h. Samples were collected for the determination of the OD_600_, dry cell weight, fibrinolytic activity, and protein concentration.

### Purification of rhμPlg

The culture was centrifuged and the supernatant was ultrafiltered, followed by gel-filtration and cation-exchange chromatography. The concentrated supernatant was loaded onto a Sephadex G-50 column (7.5 cm × 100 cm) that was already equilibrated with 20 mmol/L acetate buffer (pH 5.0). One thousand milliliters of collected sample, which was eluted from the gel-filtration column, was loaded onto a SP-Sepharose FF column (2.6 cm × 20 cm), which was also equilibrated with 20 mmol/L acetate buffer (pH 5.0). It was washed with 20 mmol/L acetate buffer (pH 5.0), followed by a single linear gradient of 0–1.0 mol/L NaCl-acetate buffer. The fractions were collected for activity assays and SDS-PAGE analysis, then desalted with a Superdex 75 column (5.0 cm × 100 cm). Forty milliliters of sample was loaded each time. The desalted sample was lyophilized and stored at −80 °C.

### Western blotting

Proteins resolved by SDS-PAGE were electrotransferred to a polyvinylidene fluoride membrane followed by blocking in 5 % skim milk prepared in Tris-buffered saline containing 0.05 % Tween-20 (TBST). The membrane was then incubated with an anti**-**rhμPlg polyclonal antibody (1:1000 dilution) (16776-1-AP, Proteintech Group, Chicago, IL, USA) for 90 min at room temperature. After washing, the membrane was incubated with a horseradish peroxidase-conjugated goat anti-rabbit IgG (1:10,000 dilution) (CW0103, Cwbiotech, Beijing, China) for 60 min at room temperature. All antibody incubations and washing steps were conducted in TBST. Immunoreactive bands were visualized with an enhanced chemiluminescent substrate (PA109, Tiangen Biotech, Beijing, China).

### Mass spectrometry analysis

The purified protein was analyzed by mass spectrometry. Molecular weight measurements were made by matrix-assisted laser desorption-ionization time of flight mass spectrometry (AB SCIEX) at the Shanghai Applied Protein Technology Co., Ltd.

### Amidolytic assays and kinetic analysis

Amidolytic activity was measured using H-D-Val-Phe-Lys-p-NA · 2HBr, a chromogenic substrate containing a ρ-nitroanilide (ρ-NA) group [32]. Human plasminogen was used as a standard to calculate the activity units of rhμPlg. Fifty microliters of substrate (1.25 mM) was added to 50-μl solutions of various concentrations of proteinases in 50 mM Tris–HCl, pH 7.4. The release of ρ-NA was then monitored continuously at 405 nm. The amount of substrate hydrolyzed was calculated from the increase in absorbance at 405 nm, using a molar extinction coefficient of 10,000 M^−1^ · cm^−1^ for free ρ-NA. One unit of enzyme will produce one umole of ρ-nitroanilide from H-D-Val-Phe-Lys-p-NA · 2HBr per minute at 37 °C, pH 7.4 (Amidase unit). The Michaelis constant (*K*_*m*_) and catalytic rate constant (*k*_*cat*_) were determined from Lineweaver–Burk plots.

### Enzyme assays and protein analysis

The catalytic activity of rhμPlg was assayed and compared with that of plasminogen. Fibrinolysis was performed on a fibrin plate, as described by Choi and Sa [33]. Briefly, a mixture of 0.6 % (w/v) fibrinogen and 2 % (w/v) agar was prepared in 50 mM sodium phosphate buffer at pH 7.4, boiled for 2 min, cooled to 55 °C, and subsequently mixed with thrombin (10 NIH units ml^−1^) for coagulation in a Petri dish. Serial dilutions of the enzyme solution (1, 2, 3, 4, and 5 U/mL) were prepared in 50 mM sodium phosphate buffer, and approximately 15 μl was injected into sample pools in the fibrin plate and incubated at 37 °C for 18 h. The product of the diameters of two perpendicular lysis zones was used as the measure of fibrinolytic activity. The areas of the zones hydrolyzed by the same concentration of protease were compared.

Total protein concentrations were determined by the BCA assay kit (Thermo Fisher Scientific, Waltham, MA, USA) using bovine serum albumin as a standard. Proteins were analyzed on 12 % polyacrylamide gels under denaturing conditions. BandScan, a software to detect protein or nucleic acid quantity by gray scanning, was used for analysis of protein expressed level under different conditions [34]. The target protein concentration was determined by scanning a stained SDS–PAGE with BandScan software. Gray scanning analysis by BandScan software showed that the target protein under different concentrations accounted for the percent of the total protein. Then the target protein concentration can be caculated. N-terminal sequencing was performed by Shanghai Genecore Biotechnologies (Shanghai, China).

## Additional files

Additional file 1:
**Cloning and schematic representation of** ***hμPlg*** **cloning.** Figure (A) represents cloning of *hμPlg* from plasmid pUC57 to pPIC9K vector. M – DNA Marker, 1- PCR with gene specific primers using pUC57 as template, 2- restriction digestion of pPIC9K-*hμPlg* with *XhoI* and *Sal*I, 3- restriction digestion of pPIC9K-*hμPlg* with *Not*I and *Sal*I, 4- restriction digestion of pPIC9K-*hμPlg* with *Rsr*II. Figure (B) represents the schematic diagrams of pPIC9K-*hμPlg*. Additional file 2:
**Summary of the purification of rhμPlg.**
Additional file 3:
**Nucleotide sequence and corresponding amino acid sequence of hμPlg.** The mature cDNA sequence of rhμPlg encodes a 261-amion acid peptide.

## Abbreviations

hμPlg: Human microplasminogen
rhμPlg: Recombinant human microplasminogen
rhμPlm: Recombinant human microplasmin
tPA: Tissue plasminogen activator
*AOX*: Alcohol oxidase
uPA: urokinase plasminogen activator
SP: Serine protease
ECM: Extracellular matrix
MD: Minimal dextrose
YPD: Yeast extract-peptone-dextrose
BMGY: Buffered complex glycerol medium
BMMY: Buffered complex methanol medium
BSM: Basal salts medium
MW: Molecular weight
TBST: Tris-buffered saline containing 0.05 % Tween-20
ρ-NA: ρ-nitroanilide
*Km*: Michaelis constant
*kcat*: Catalytic rate constant
SDS-PAGE: Sodium dodecyl-sulfate polyacrylamide gel electrophoresis
