# Supplementary material for: High-level expression, purification, and enzymatic characterization of truncated human plasminogen (Lys531-Asn791) in the methylotrophic yeast Pichia pastoris
Source: BMC Biotechnol. 2015 Jun 9;15:50. doi: 10.1186/s12896-015-0179-z (PMC4460660; doi:10.1186/s12896-015-0179-z)
Supplement: Additional file 1: — Cloning and schematic representation of hμPlg cloning. Figure (A) represents cloning of hμPlg from plasmid pUC57 to pPIC9K vector. M – DNA Marker, 1- PCR with gene specific primers using pUC57 as template, 2- restriction digestion of pPIC9K-hμPlg with XhoI and SalI, 3- restriction digestion of pPIC9K-hμPlg with NotI and SalI, 4- restriction digestion of pPIC9K-hμPlg with RsrII. Figure (B) represents the schematic diagrams of pPIC9K-hμPlg. [file 12896_2015_179_MOESM1_ESM.docx]

**Additional file 1: Cloning and schematic representation of *hμPlg* cloning.**


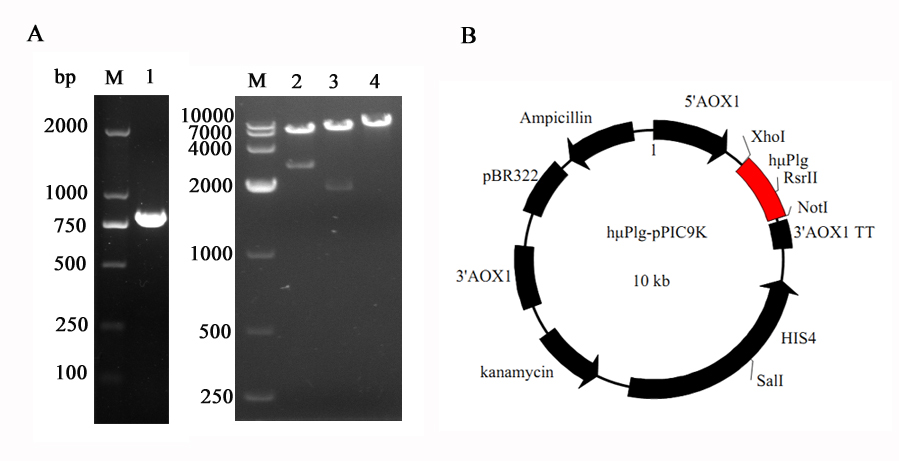


**Additional file 1: Cloning and schematic representation of *hμPlg* cloning.** Figure (A) represents cloning of *hμPlg* from plasmid pUC57 to pPIC9K vector. M – DNA Marker, 1- PCR with gene specific primers using pUC57 as template, 2- restriction digestion of pPIC9K-*hμPlg* with *Xho*I and *Sal*I, 3- restriction digestion of pPIC9K-*hμPlg* with *Not*I and *Sal*I, 4- restriction digestion of pPIC9K-*hμPlg* with *Rsr*II. Figure (B) represents the schematic diagrams of pPIC9K-*hμPlg*.
