# Supplementary material for: High-level expression, purification, and enzymatic characterization of truncated human plasminogen (Lys531-Asn791) in the methylotrophic yeast Pichia pastoris
Source: BMC Biotechnol. 2015 Jun 9;15:50. doi: 10.1186/s12896-015-0179-z (PMC4460660; doi:10.1186/s12896-015-0179-z)
Supplement: Additional file 2: — Summary of the purification of rhμPlg. [file 12896_2015_179_MOESM2_ESM.docx]

**Additional file 2. Summary of the purification of rhμPlg**

| **Purification steps** | **Total protein (mg)** | **Total activity**  **(U)** | **Specific activity (U.mg^-1^)** | **purification (fold)** | **Yield(%)** |
| --- | --- | --- | --- | --- | --- |
| The supernatant | 3400 | 26860 | 7.9 | 1 | 100 |
| 5 kDa ultrafiltrate | 1842 | 19715 | 10.7 | 1.4 | 54.2 |
| Sephadex G-50 | 1380 | 18157 | 13.2 | 1.7 | 40.6 |
| SP Sepharose FF | 781 | 15549 | 19.9 | 2.5 | 22.9 |
| Superdex 75 | 568 | 13400 | 23.6 | 3.0 | 16.7 |
