# Supplementary material for: High-level expression, purification, and enzymatic characterization of truncated human plasminogen (Lys531-Asn791) in the methylotrophic yeast Pichia pastoris
Source: BMC Biotechnol. 2015 Jun 9;15:50. doi: 10.1186/s12896-015-0179-z (PMC4460660; doi:10.1186/s12896-015-0179-z)
Supplement: Additional file 3: — Nucleotide sequence and corresponding amino acid sequence of hμPlg. The mature cDNA sequence of rhμPlg encodes a 261-amion acid peptide. [file 12896_2015_179_MOESM3_ESM.pdf]

```
Translation of hμPlg.seq(1-786)
Universal code
Total amino acid number: 261, MW=28580
Max ORF starts at AA pos 1(may be DNA pos 1) for 261 AA(783 bases), MW=28580
                  10        20        30        40        50        60
1         AAACTTTACGACTACTGTGATGTCCCTCAGTGTGCGGCCCCTTCATTTGATTGTGGGAAG
1          K  L  Y  D  Y  C  D  V  P  Q  C  A  A  P  S  F  D  C  G  K

                  70        80        90       100       110       120
61        CCTCAAGTGGAGCCGAAGAAATGTCCTGGAAGGGTTGTGGGGGGGTGTGTGGCCCACCCA
21         P  Q  V  E  P  K  K  C  P  G  R  V  V  G  G  C  V  A  H  P

                 130       140       150       160       170       180
121       CATTCCTGGCCCTGGCAAGTCAGTCTTAGAACAAGGTTTGGAATGCACTTCTGTGGAGGC
41         H  S  W  P  W  Q  V  S  L  R  T  R  F  G  M  H  F  C  G  G

                 190       200       210       220       230       240
181       ACCTTGATATCCCCAGAGTGGGTGTTGACTGCTGCCCACTGCTTGGAGAAGTCCCCAAGG
61         T  L  I  S  P  E  W  V  L  T  A  A  H  C  L  E  K  S  P  R

                 250       260       270       280       290       300
241       CCTTCATCCTACAAGGTCATCCTGGGTGCACACCAAGAAGTGAATCTCGAACCGCATGTT
81         P  S  S  Y  K  V  I  L  G  A  H  Q  E  V  N  L  E  P  H  V

                 310       320       330       340       350       360
301       CAGGAAATAGAAGTGTCTAGGCTGTTCTTGGAGCCCACACGAAAAGATATTGCCTTGCTA
101        Q  E  I  E  V  S  R  L  F  L  E  P  T  R  K  D  I  A  L  L

                 370       380       390       400       410       420
361       AAGCTAAGCAGTCCTGCCGTCATCACTGACAAAGTAATCCCAGCTTGTCTGCCATCCCCA
121        K  L  S  S  P  A  V  I  T  D  K  V  I  P  A  C  L  P  S  P

                 430       440       450       460       470       480
421       AATTATGTGGTCGCTGACCGGACCGAATGTTTCATCACTGGCTGGGGAGAAACCCAAGGT
141        N  Y  V  V  A  D  R  T  E  C  F  I  T  G  W  G  E  T  Q  G

                 490       500       510       520       530       540
481       ACTTTTGGAGCTGGCCTTCTCAAGGAAGCCCAGCTCCCTGTGATTGAGAATAAAGTGTGC
161        T  F  G  A  G  L  L  K  E  A  Q  L  P  V  I  E  N  K  V  C

                 550       560       570       580       590       600
541       AATCGCTATGAGTTTCTGAATGGAAGAGTCCAATCCACCGAACTCTGTGCTGGGCATTTG
181        N  R  Y  E  F  L  N  G  R  V  Q  S  T  E  L  C  A  G  H  L

                 610       620       630       640       650       660
601       GCCGGAGGCACTGACAGTTGCCAGGGTGACAGTGGAGGTCCTCTGGTTTGCTTCGAGAAG
201        A  G  G  T  D  S  C  Q  G  D  S  G  G  P  L  V  C  F  E  K

                 670       680       690       700       710       720
661       GACAAATACATTTTACAAGGAGTCACTTCTTGGGGTCTTGGCTGTGCACGCCCCAATAAG
221        D  K  Y  I  L  Q  G  V  T  S  W  G  L  G  C  A  R  P  N  K

                 730       740       750       760       770       780
721       CCTGGTGTCTATGTTCGTGTTTCAAGGTTTGTTACTTGGATTGAGGGAGTGATGAGAAAT
241        P  G  V  Y  V  R  V  S  R  F  V  T  W  I  E  G  V  M  R  N


781       AATTAA
261        N  *
```
